# Cardiovascular Stents: Types and Future Landscape

**DOI:** 10.7759/cureus.43438

**Published:** 2023-08-13

**Authors:** Rohit A Sahu, Aparna Nashine, Abhay Mudey, Shreya A Sahu, Roshan Prasad

**Affiliations:** 1 Community Medicine, Jawaharlal Nehru Medical College, Datta Meghe Institute of Higher Education and Research, Wardha, IND; 2 Obstretics and Gynecology, Jawaharlal Nehru Medical College, Datta Meghe Institute of Higher Education and Research, Wardha, IND; 3 Medicine and Surgery, Jawaharlal Nehru Medical College, Datta Meghe Institute of Higher Education and Research, Wardha, IND

**Keywords:** heart disease, cardiovascular intervention, carotid blood flow, biomedical applications, coronary stents

## Abstract

One of the prominent reasons for mortality and morbidity worldwide is coronary artery disease (CAD), an ailment that manifests itself by the narrowing of the artery with the deposition of plaque. The definitive mode of action for dealing with this condition is using a medical device known as a stent at the affected location. This extremely important tubular equipment helps tremendously with vessel support. It also helps by keeping the path of blood flow clear for the heart muscle masses, its crucial nutrients, and oxygen supply. Several generations of stents have been continuously developed to improve patient outcomes and reduce side effects post-stent implantation. As we move from bare metal stents (BMSs) to drug-eluting stents (DESs) and, more recently, to bioabsorbable stents, the research area continues to develop. The use of this biomedical device has increased the standard of living in many cases; therefore, it is much needed to work on the possible growth areas in the cardiovascular stents and improve them to such an extent that the patients suffering from cardiovascular ailments get to live a comfortable life. Most articles deal with stents that are available for current use and their various types. They also cover the topic of stent optimization, as it is one of the key factors in enhancing stent usability and plays a prominent role in optimizing stent placement in the vessels of the body. To keep in touch with advances in stent technology over the past few decades, this article reviews advances in the devices, working on how available stents can be optimized to create new stents.

## Introduction and background

Cardiovascular ailments and disorders have become a tremendously significant threat to humankind; they are the dominating cause of death globally and a major cause of hospitalization [1]. Coronary artery disease (CAD) accounts for approximately 610,000 deaths annually (estimated one in four deaths) and is the leading cause of mortality in the United States. It is responsible for 17.8 million deaths annually and is the third biggest cause of mortality worldwide [2]. It causes tremendous economic and health impediments in some of the most progressive nations [3]. The narrowing of the artery portrays CAD, the reason being plaque precipitating under the endothelium. Cells, fat, calcium, cellular waste, and other materials can amass in these deposits, triggering a stream of events that reduces the lumen of the blood vessels. It reduces blood outpouring and leads to an improper supply of nutrients and oxygen to the heart muscle, which can eventually lead to myocardial infarction, heart attack, or transient cerebral ischemic stroke and attacks [4].

Inserting a stent into the blood vessel under endoscopic or fluoroscopic guidance aims to bring back regular blood flow and prevent other significant vasoconstriction outcomes. This technique is less invasive than open heart surgery, has lower long-term mortality and morbidity in critically ill patients, and has promising short-term outcomes [5]. A study was done by Diegeler et al. where he compared stenting with minimally invasive bypass surgery for stenosis of the left anterior descending coronary artery. They concluded that a major adverse cardiac event occurred in 31% of patients after stenting, compared with 15% in the surgery group (P=0.02). Moreover, adverse events occurred more frequently after surgery [6].

As such, stents are the major life-saving instruments and thus are ranked in the top 10 medical advances of the century. Structurally, the stent is a tiny, sophisticated, cylindrical indented structure shaped into a continuous coil structure containing a strut in a series and connectors. The working of each stent depends on its design. This helps keep the arterial pathways in the human body open throughout the body [5]. Initially, stents made of bare metals were used as an emergency intervention in cases of sudden and threatening vascular occlusion with balloon angioplasty, which has become an outdated practice these days.

Positive results have contributed to the increased acceptance of stent implantation as the primary criterion for percutaneous coronary intervention (PCI). Nonetheless, the clinical defence and effectiveness of using first-generation devices are often restricted to intra-stent restenosis, ensuing the oversight of occurring stents or re-entry with other stents [7]. A few years later, one solution to this problem, the position of drug-releasing or drug-eluting stents (DESs), became an essential option for treating such patients with CAD. Coronary DES has also been adopted as the recent standard because it considerably reduces the restenosis of vascular stents and reduces the demand for repeat revascularization [8]. Years of analysis and examinations in this field have led to various stent layouts using various materials that allow clinicians to choose the device appropriate based on fatty deposits and local plaque and side effects that can be seen. Therefore, this review aims to evaluate past and presently available stents and elucidate the working of these devices for their further optimization to achieve an ideal in vivo working.

## Review

Methodology

**Figure 1:**
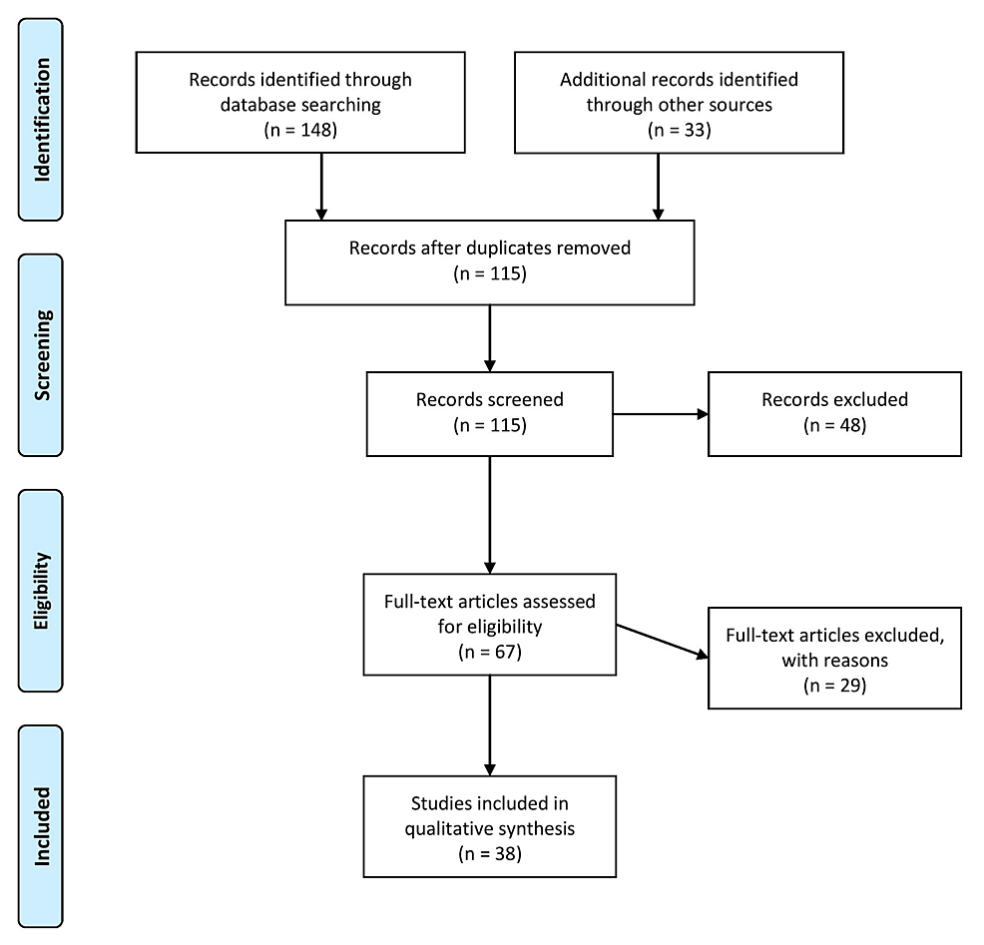
Selection process of articles used in this study. Adopted from the Preferred Reporting Items for Systematic Reviews and Meta-Analyses (PRISMA)

A comprehensive review of the evolution of cardiovascular stents with time, their different types available nowadays, and future possibilities was conducted using a comprehensive literature search. Relevant articles were identified by searching electronic databases, such as PubMed, Scopus, and Web of Science, using keywords and combinations related to heart disease, cardiovascular intervention, carotid blood flow, biomedical applications, and coronary stents. No language or time restrictions were applied, and original research articles and review papers were considered. In addition, reference lists of retrieved articles were examined to identify any additional relevant studies. The inclusion criteria involved selecting studies relevant to the topic. Only peer-reviewed articles published in Scopus, Web of Science, or PubMed indexed journals were included to ensure quality and validity. The screening process initially assessed titles and abstracts, followed by a thorough evaluation of full-text articles based on the inclusion and exclusion criteria. The exclusion criteria required that the articles be published in a language other than English and in non-peer-reviewed journals. Any uncertainties or discrepancies in the study selection were resolved through reviewer discussions involving two reviewers. By applying these rigorous criteria, the selected studies incorporated in this review article are reliable and pertinent to the topic at hand. Figure 1 describes the selection process of articles used in our study.

Cardiovascular stents and their evolution with time

Rapid technological advances over the past 40 years have had a major impact on the advancement of PCI. There has been an evolution in interventional cardiology since the first surgery conducted in regard to percutaneous coronary angioplasty [9]. The evolution started with a balloon catheter attached to an anchoring wire; progressed to first-generation DES, bare metal stents (BMS), second-era biodegradable polymeric DES, and third-era biodegradable polymeric DES; and culminated with the introduction of bioabsorbable vascular scaffolds. Bioresorbable vascular stents (BVSs) are currently under development. Stents have been found to carry universal medical uses in various emergency situations since the beginning of their use in the United States in the 1960s by Charles Theodore Dotter, but their true potential use in CAD has yet to be discovered. Two interventions were introduced: coronary angiography and balloon angioplasty [10]. The arterial recoil and vascular dissection rate was high in simple balloon angioplasty without initial stent insertion.

Different types of stents

BMSs

The initial coronary stents were introduced in 1986 and served as the starting juncture for the first-generation susceptibility gradient mapping (SGM). These are typically made of corrosion-resistant substances, such as stainless steel 316L, cobalt-chromium (Co-Cr), tantalum (Ta), nitinol (Ni-Ti), or platinum-iridium (Pt-Ir), and can be permanently implanted to work throughout life [11]. A common problem in stent design is recoil exhibition, which indicates a proportion reduction in the stent diameter between the widened and shortened configurations. The stent material must have appropriate mechanical properties to avoid significant dimensional changes. The common fabrication procedure for all substances is laser slicing, but nitinol also uses thin-film technology to process it into a stent, followed by photo etching [12]. Although stainless steel is a tough, non-corrosive, and hard substance, it has serious drawbacks regarding clot formation due to its limited biocompatibility. Nitinol alloys, on the other spectrum, have shown better biocompatibility in the small span, but in due course of time, nickel may leave out of the substance used and cause issues related to immune response. Tantalum metal is non-ferromagnetic and stable, and the surface layer of oxide formed after implantation is considered biocompatible. However, there was no difference in the rate of clot formation when this material was compared with stainless steel [13].

Drug-Releasing Stents

One of the important turning stones in the sector of interventional cardiology was when the first DES was brought to market in 2002. The layout of the first-generation DES was based on a stainless steel staging covered with a strong drug-releasing polymer. These devices allow localization within one month of neointimal inhibitory medications, such as sirolimus and paclitaxel, which possess anti-proliferative effects [14]. DES has been widely accepted as a remedy to stent restenosis, an important difficulty linked with stenting. The tested long-term protection and usefulness are also recognized in a higher rate of subsequent vascular regeneration and reduced danger of thrombosis approximated to BMS. However, the initially developed DES has a prolonged duration of dual antiplatelet therapy (DAPT) instead of SGM, primarily accompanied by increased bleeding [15]. The major risk of stent thrombosis, especially in SGM, peaks during the first 14-30 days. Therefore, while DAPT is generally suggested for the short duration of one month, a period of a minimum of six to 12 months is required after DES transplantation to prevent late stent thrombosis. To prove the point, both observational and randomized investigations have shown a constant growth in the total occurrence of late stent thrombosis. By contrast, pathological research has demonstrated that strong polymers used primarily as drug carriers prolong tissue inflammation around the stent, delaying arterial healing [16].

Bioabsorbable Stents

Stents are usually needed for a short duration of time that is temporary (up to healing and reendothelialization) because of their short-time usefulness, but during the long course of time, they are seen to cause serious complications related to metal residues. Next-generation instruments (characterized in the writings as bioabsorbable and biodegradable stents) are presently generated to decrease side effects, such as restenosis, chronic inflammation, progressive thrombosis, and vessel size mismatch [17]. The introduction of bioabsorbable stents is deemed a major advancement in the field of interventional cardiology. These instruments are made of substances that provide temporary assistance and, after the remodelling process, dissolve or absorb into the body and degrade gradually. In this regard, there is a growing interest in biodegradable polymers and biocompatible and metal-made materials. Production methods include photochemical etching, spiral winding, weaving, liquid injection, three-dimensional (3D) printing, hot extrusion, selective laser melting, and electrospinning [18,19]. Of these, poly-L-lactide acid (PLLA) was used in the first BVS approved by the European Medicines Agency and prevails as the vastly representative biocompatible polymer utilized in new bioabsorbable stents, Poly-L-lactide (PLA) is also one such biodegradable polymer that possesses satisfactory technical and .mechanical properties, which can be used in polymer blends or singularly for the manufacture of next-generation stents [20]. Different polymers that can be reabsorbed in the body within a few months of stenting include polyglycolic acid (PGA), poly(lactic-co-glycolic acid) (PLGA), polycaprolactone (PCL), phosphoryl choline, polyorthoester, hyaluronic acid, fibrin, polybutylene terephthalate, and polyethylene oxide.

Stent optimization

For any stent to be coined as ideal, it must have flexibility, biocompatibility, delivery ability, good radiopacity under fluoroscopy, and strong radial force. A favorable instrument should furthermore have a poor incidence of thrombus formation, neointimal proliferation, and stent thrombosis at long-standing follow-up [21]. For the best treatment of CAD, the stent should not come in contact with active restenosis drugs, discharge the medication at an appropriate amount, be biologically inactive, and be mechanically dependable for an extended period of time. The stent should inflict the least amount of injury to the vascular wall, cause the least provocative response, be adequately reendothelialized, contribute to a vascular skeleton, and ultimately facilitate vascular recovery and remodelling. The many conflicting requirements presented above fall into three main categories in the literature, each with several key criteria, such as transmissibility, efficacy, and safety [22,23].

Contemporary platforms

Several recent stent platforms are in the development process or clinical trials. Researchers have shown that the shape of the stent is a determinant of restenosis. Assuming that the substance and composition do not change, a boost in the amount of assistance struts leads to a proportionate improvement in the neointimal region, thereby decreasing vascular damage. An additional instance of structure advancement is the dealings of bifurcated stents that can withstand the challenge encountered in bifurcation procedures. Bifurcated PCI is linked with a high incidence of perioperative drawbacks, intra-stent restenosis, and stent thrombosis [24]. An assigned bifurcated stent can be selected for this method because the surgeon can place a stent in the lesion without rewiring the side area. This substitute for a complicated two-stent strategy yielded clinical outcomes similar to the traditional interim stent placement approach. Not only can the design of the stent that can be enhanced even newer substances are being studied. One such metal specific is zinc. It has captivated a lot of interest for use in biomedical implants. Although zinc-based materials have not yet been actively used for bioabsorbable stents, they are considered superior to magnesium alloys [25]. They possess ideal in vivo degradation rates (automatic integrity is retained for six months post-transplantation, and about 50% degradation is maintained 12 months post-transplantation), good overall biocompatibility, low smooth muscle cell proliferation, and excellent antibacterial effects. Zinc also has more ductility than magnesium, making these alloys easier to machine into desired designs. The span of properties (mechanical) achieved by zinc alloys meets the requirements of cardiovascular stents. This is because particular substances have tensile strengths from 87 to 399 megapascals (MPa) and a lengthening at fracture values of 0.9% to 170%. Some other setting entities that are experimented with for bioabsorbable stents comprise ferrous alloys. Because iron decomposes slowly, mixing it with different metals in the right proportions speeds up the corrosion without compromising the mechanical properties required. In particular, positive outcomes have been gained using Fe-35Mn (a ferrous alloy constituting approximately 35% manganese) with the same yield strength and tensile as stainless steel but a decomposition rate of three to eight times faster [26-28].

Surface modifications

Another approach to enhancing stents' safety, biocompatibility, and effectiveness is modifying the exterior of the implant. Biological reactions can be enhanced by three categories of surface modifications: physicochemical, topographical, and surface biofunctionalization. Topographical changes infer the production of precise micro- or nanostructured coverings to facilitate endothelium healing. The main principle behind this technique is to get a monitored biomimetic outline that can improve endothelial cell migration and adhesion on the stent surface [29]. The depth of the pattern should be in the submicrometer spectrum to prevent undesirable platelet attachment. Several types of exterior solutions, such as low-pressure plasma etching, electro-polishing, chemical etching, ultrasonic cleaning, degreasing, and motorized polishing, can be assigned to the stent platform to achieve a smooth surface without any problems. Moreover, endothelial distribution and adhesion can be altered through certain physicochemical modifications. This can be accomplished by creating appropriate working groups or controlling surface endurance. For this very reason, metal nitrides and oxides can be utilized [30]. However, relatively, polymers and metals can be inserted using various physicochemical methods, including pulsed laser deposition, magnetron sputtering, and pulsate laser evaporation (matrix-assisted). An additional instance of chemical improvement of a stent surface is its precipitate on a molecular layer of silane, a compound rich in beneficial functional groups. An additional important example of exterior modification is biofunctionalization, which pertains to immobilization on the exterior of biomolecules with certain distinct natural properties while simultaneously maintaining the actual mechanical properties of the substance. Cell-substance exchanges can be enhanced with biomacromolecules (e.g., heparin, chondroitin sulphate, fucoidan, antioxidant compounds, hyaluronic acid, or collagen) that promote a sequence of occurrences constructive to the regeneration of injured areas with active endothelium [31-34].

Future prospectives

At present, stents are primarily generated by beam cutting or different manufacturing methods, such as waterjet cutting, electrode discharge machining, photochemical tube etching, and various wire forming methods, including knitting and braiding [19]. However, these procedures can be upgraded or renovated with advances in 3D printing, augmented reality (AR), and deep learning (DL). In particular, using the blood vessel information collected via DL and AR, entities, such as PLA, polydiol citrate, and metallic glasses, can be 3D printed on cardiovascular appliances with a clearer design than functional ones on the market that can be printed [5]. Using these technologies opens up the possibility of patient-particular devices that can be precisely adjusted to each individual's necessities [19]. Thus, the issues of inflammation, immunogenicity, fibrous tissue formation, cytotoxicity, and material degradation can be dealt with by creating individual cardiovascular stents corresponding to the target vessel's pathological and physiological conditions. Introducing a smart stent instead of a basic support device is also possible. Researchers have also proposed an innovative biocompatible and implantable platform to measure blood flow using a small ultrasound transducer [35,36]. These systems provide flexibility because they can receive the information wirelessly. Thus, smart stents can prohibit restenosis while controlling post-implant consequences in situ. The succeeding path for the future is the substitute of the basic conventional implantation technique. Another future direction is to replace traditional implantation procedures. With their success in delivering drugs through blood vessels with biocompatible, high-precision 3D-printable micro-robots, Zurich developers began exploring microrobots for stent placement. The exact university is also introducing new four-dimensional (4D) printing techniques to produce cardiovascular stents that are 40 times smaller than currently available ones [37,38].

## Conclusions

Cardiovascular diseases pose a severe danger to a large fraction of the earth's population and affects both characteristics and life expectancy. In particular, arterial stenosis due to plaque deposition is followed by a series of exacerbated events. Each stage of technological advances in coronary stenting has impacted percutaneous coronary interventions, improving small and long-term outcomes. DESs have become the ideal care in PCI because the DES platform includes crucial improvements in polymer, scaffold design, proliferative drug delivery, and compatibility. In addition, recently bioabsorbable stents have become a convenient solution when a permanent stent is required because degradation prevents undesirable long-term consequences. However, despite the safety and effectiveness of newer equipment pertained to previous ages, difficulties can still arise and some people suffer from stent letdown, restenosis, and thrombosis. Consequently, there is a need to explore modern materials for both stent outlets and polymer coverings and develop more advanced stent configurations. As such, although stents have been used for a long period of time, they still possess significant analysis potential. Stent optimization must be accomplished at every step pertained to, from composition, material selection, and manufacturing methods to surface implantation procedures and functionalization.
